# Fluxomics - New Metabolomics Approaches to Monitor Metabolic Pathways

**DOI:** 10.3389/fphar.2022.805782

**Published:** 2022-03-21

**Authors:** Abdul-Hamid Emwas, Kacper Szczepski, Inas Al-Younis, Joanna Izabela Lachowicz, Mariusz Jaremko

**Affiliations:** ^1^ King Abdullah University of Science and Technology, Core Labs, Thuwal, Saudi Arabia; ^2^ Smart-Health Initiative (SHI) and Red Sea Research Center (RSRC), Biological and Environmental Sciences & Engineering Division (BESE), King Abdullah University of Science and Technology (KAUST), Thuwal, Saudi Arabia; ^3^ King Abdullah University of Science and Technology (KAUST), Biological and Environmental Sciences & Engineering Division (BESE), Thuwal, Saudi Arabia; ^4^ Department of Medical Sciences and Public Health, University of Cagliari, Cittadella Universitaria, Monserrato, Italy

**Keywords:** fluxomics, metabolomics, nuclear magnetic resonance (NMR), mass spectrometry (MS), flux, pharmacometabolomics

## Abstract

Fluxomics is an innovative -omics research field that measures the rates of all intracellular fluxes in the central metabolism of biological systems. Fluxomics gathers data from multiple different -omics fields, portraying the whole picture of molecular interactions. Recently, fluxomics has become one of the most relevant approaches to investigate metabolic phenotypes. Metabolic flux using ^13^C-labeled molecules is increasingly used to monitor metabolic pathways, to probe the corresponding gene-RNA and protein-metabolite interaction networks in actual time. Thus, fluxomics reveals the functioning of multi-molecular metabolic pathways and is increasingly applied in biotechnology and pharmacology. Here, we describe the main fluxomics approaches and experimental platforms. Moreover, we summarize recent fluxomic results in different biological systems.

## Introduction

Throughout recent decades discoveries explaining the complex nature of the cell have provided the scientific community with an immense amount of data. As more information has been revealed, the need for classification and quantification of this data has resulted in the creation of various–omics fields. This approach recognizes whole systems, rather than groups of separated processes (Vailati-Riboni et al., 2017). Many types of–omics have been created, the most prominent being genomics, transcriptomics, proteomics and metabolomics. All of these fields are part of systems biology - a strategy used to examine the interactions, relationships and behavior between all system constituents (Ideker et al., 2001). However, even though the fundamental–omics approaches focus only on their system of interest (e.g. the genome for genomics, or the proteome for proteomics), their constituents are connected. For example, the field of proteomics exists as the directional effect of transcriptomics that is further influenced by genomics.

**FIGURE 1:**
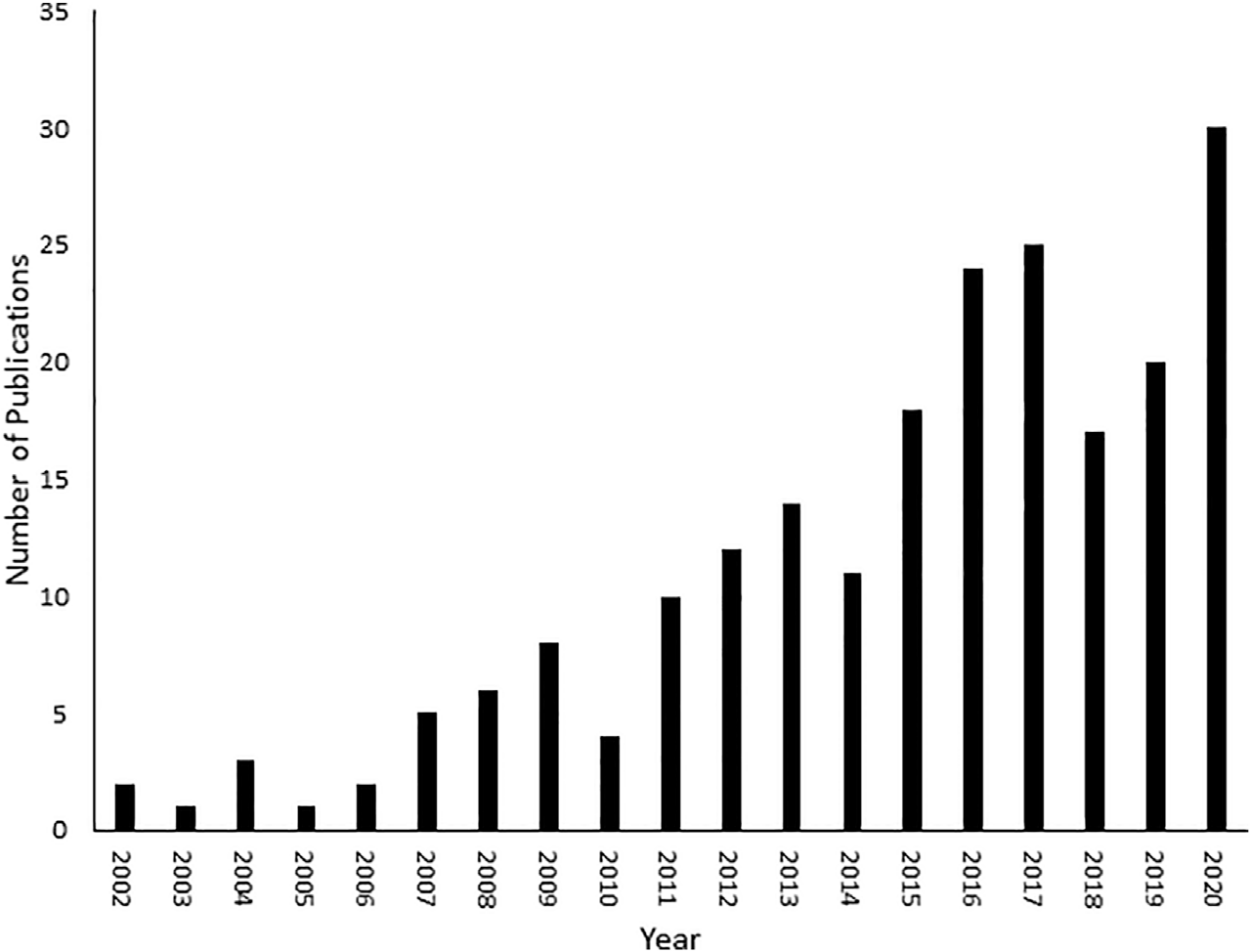
Number of fluxomic publications. A literature review was conducted on SciFinder (https://scifinder.cas.org/scifinder/view/scifinder/scifinderExplore.jsf) using the keyword fluxomics.

Given these factors, a new discipline called fluxomics emerged that connects genomics, transcriptomics, proteomics and metabolomics. Although a new addition to the–omics family, fluxomics studies have been steadily increasing over the past 2 decades (Figure 1). Recent examples of fluxomics studies are shown in Supplementary Table S1. The emerging importance of fluxomics is reflected not only by the amount of research articles published every year but also through its potential applications in industrial biotechnology and pharmacology (Feng et al., 2010; Wojtowicz and Mlynarz, 2016; Hansen et al., 2017; Emwas, 2021). Several recent studies used fluxomics as an alternative approach in the field of drug discovery, by targeting bacterial metabolic pathways distinct from human metabolic routes. Viral and bacterial infection depends on the ability of pathogens to convert nutrients into energy (e.g., ATP) (Eisenreich, 2021). Importantly, bacteria have partially distinct metabolic pathways compared to their human host cells (Rohmer et al., 2011). Selective inhibition of differential mechanisms is unlikely to have major side effects in humans. Innovative drug therapies that reprogram the core carbon metabolism of human infections make bacteria more susceptible to antibiotics (Liu et al., 2019a; Stokes et al., 2019). A recent study examined the metabolomic profile of *Vibrio alginolyticus*, which is resistant to cephalosporin antibiotics, and the role of bacterial metabolism in drug and multidrug resistance. This was achieved by detecting the metabolic differences of acetyl-CoA fluxes into and through the P-cycle and fatty acid biosynthesis (Liu et al., 2019b). These findings shed light on ceftazidime (CAZ) and other antibiotic resistance pathways, as well as multidrug resistance of *Vibrio* and other pathogens. A combined metabolomics and fluxomics approach was used in studies of *Leishmania infantum promastigotes*. The origin of the detected alterations was analyzed with untargeted analysis of metabolic snapshots (of treated and untreated parasites), both resistant and responders, and by using a ^13^C traceability experiment (Rojo et al., 2015). This showed a significant shift in amino acid metabolism, and multi-target metabolic change as a result of treatment, particularly affecting the cell redox system, which is critical for detoxification and biosynthetic activities (Rojo et al., 2015). Although there are costs and current challenges associated with fluxomics approaches, there have been studies supporting its use in models such as *Escherichia coli* **,** *Bacillus subtilis*, *Saccharomyces cerevisiae* or *Pichia pastoris* (Feng et al., 2010; Zahrl et al., 2017). These studies provided information such as optimal fermentation conditions, improved ethanol and riboflavin production and better yield in protein expression (Feng et al., 2010; Zahrl et al., 2017; Choi et al., 2018). The constant development and improvement of analytical tools and methodologies in fluxomics will only increase its future prevalence (Wiechert et al., 2001; Beyß, 2019; Foguet et al., 2019; Giraudeau, 2020).

## Advantages and Disadvantages of Chromatography and Nuclear Magnetic Resonance Tools in Fluxomics

Similar to other -omics fields, fluxomics is a technology driven field where recent advances in instrumentation, software and databases have significantly contributed to development. Different analytical tools and approaches in fluxomics have been reviewed recently (Wiechert et al., 2007; Niittylae et al., 2009; Klein and Heinzle, 2012; Winter and Krömer, 2013; Niedenführ et al., 2015). Even if different analytical tools are utilized in fluxomics/metabolomics research, nuclear magnetic resonance (NMR) spectroscopy (Giraudeau, 2020) and mass spectrometry (MS) (Wiechert et al., 2007; Choi and Antoniewicz, 2019; Babele and Young, 2020) are the most commonly used tools in metabolomic studies.

Each applied analytical platform, either NMR or MS, has its strength, advantages and limitations. For example, gas chromatography-mass spectrometry (GC-MS) is commonly used in fluxomics analyses but is only applicable for volatile metabolites or ones that can be treated to become volatile compounds through derivatization processes. Liquid chromatography-mass spectrometry (LC-MS) provides potent approaches that offer combined sensitivity and selectivity. MS approaches such as different ionization modes (positive or negative) or mass analyzer technology can be used to increase the number of detected metabolites. Nevertheless, chromatographic experiments require specific sample pre-treatment, have limited experimental time scales, and do not depict the 3D structure or interactions of the molecule.

Beside its exceptionally high sensitivity, mass spectrometry is usually combined with other powerful analytical platforms, mainly gas chromatography (GC) or liquid chromatography (LC), bringing powerful advantages that can overcome both peak overlaps and the low sensitivities of NMR approaches (Kvitvang et al., 2014; Kvitvang and Bruheim, 2015; Lien et al., 2015; Sá et al., 2017).

NMR is a non-destructive, non-selective and fast method that has been widely used for molecular identification and structural elucidation used with minimal sample preparation requirements (Atiqullah et al., 2015; Alahmari et al., 2019; Dhahri et al., 2020). While the sample is placed in a static magnetic field, it can be recovered for future analysis using other techniques and it is possible to obtain spectral results regarding how molecules move, flex, react, appear/disappear, or bind with other molecules over several time scales, providing an optimum approach for fluxomics (Blindauer et al., 1997; Wolak et al., 2012; Nargund et al., 2013; Davaasuren et al., 2017).

Thanks to the unique features briefly mentioned above, NMR is one of the main analytical techniques in metabolomics, and as such it is crucial to accurately highlight its advantages and limitations for different metabolomics applications (Emwas et al., 2019). NMR spectroscopy, particularly hydrogen detection NMR (commonly referred to as proton or ^1^H-NMR spectroscopy) can be inherently quantitative, providing a potent analytical tool for metabolomics studies (Dona et al., 2016; Markley et al., 2017). In comparison to other analytical platforms such as GC-MS and LC-MS (Ciborowski et al., 2012; Guo et al., 2013; Raji et al., 2013; Liu et al., 2014), NMR does not require extra steps for sample preparation or metabolite isolation prior to measurement, such as chromatographic separation and/or chemical derivatization. On the other hand, spectral overlap and low signal sensitivity are still the main limitations of NMR approaches, and detection of metabolites at very low concentrations is still beyond the capability of even the most sensitive NMR technologies (Emwas and Bjerrum, 2015).

Even if NMR spectroscopy offers indisputable advantages, low sensitivity is still its main limitation in fluxomic research (Emwas et al., 2013; Clendinen et al., 2014; Emwas et al., 2016; Giraudeau, 2020). Overlapping of peaks is also a major challenge in peak assignment, limiting the number of metabolites that can be identified by NMR spectroscopy (Emwas et al., 2018; O’Rourke et al., 2018; Giraudeau, 2020). The sensitivity of NMR spectroscopy has been improved significantly by dynamic-nuclear polarization (DNP) (Ardenkjær-Larsen et al., 2003; Emwas et al., 2008; Ludwig et al., 2010), cryo-probes, ultra-high magnetic fields (Deborde et al., 2017; Emwas et al., 2019), and the development of new faster methods. However, sensitivity remains a main limitation in the field (Emwas et al., 2019; Robertson et al., 2020; Chandra et al., 2021). For instance, secondary metabolites (usually existing at very low concentrations) are beyond the detection limit of NMR spectroscopy, while for volatile molecules can be detected by GC-MS combined with the mass spectrum and retention time (Emwas et al., 2015; Kohlstedt and Wittmann, 2019). Thus, integrating NMR spectroscopy with MS methods is important to give more comprehensive analysis (Fan et al., 2014; Elbaz et al., 2015; Emwas and Bjerrum, 2015; Sá et al., 2017; Bergès et al., 2021).

## Fluxomics

**FIGURE 2:**
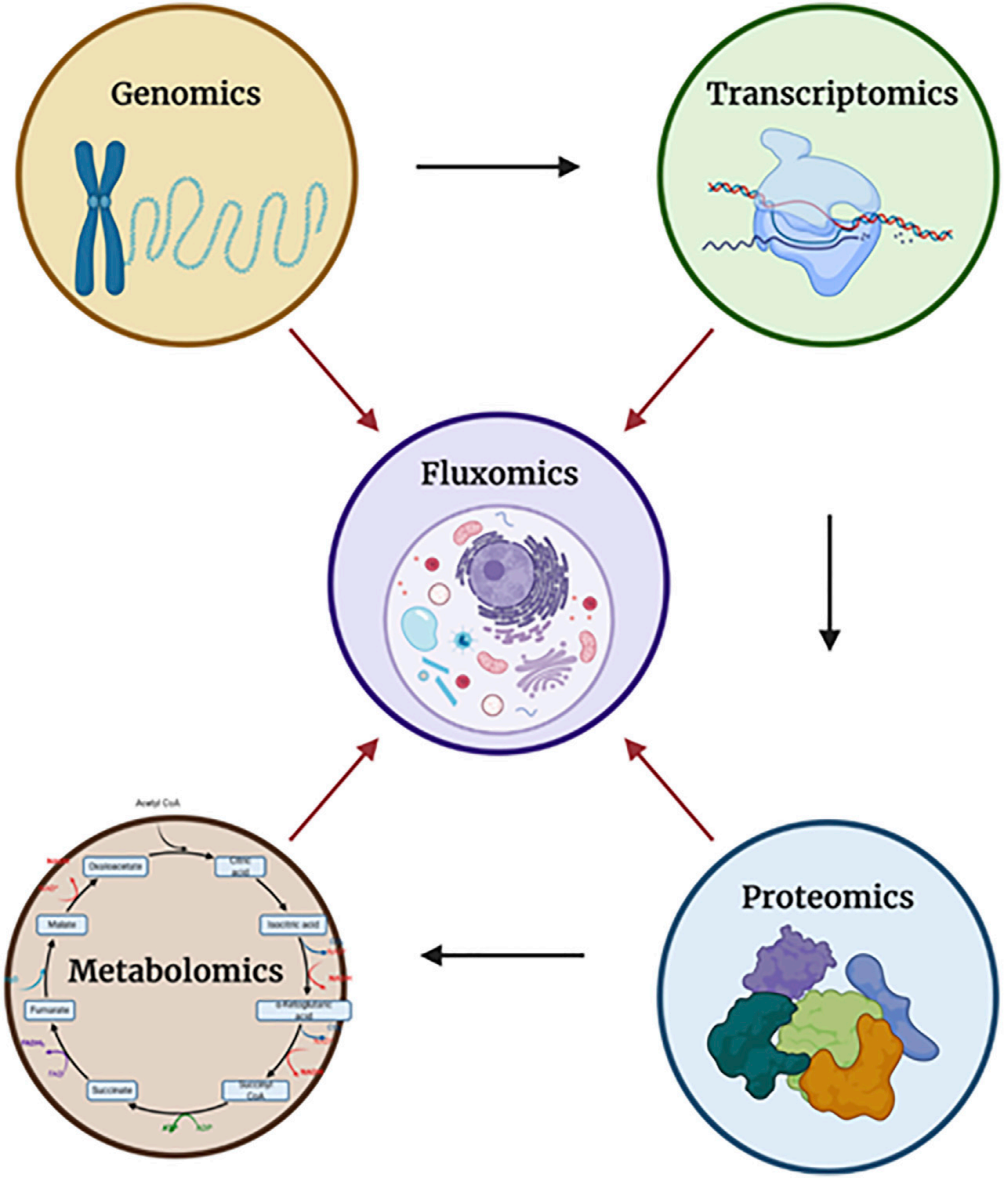
The relationships between each of the “-omics”. Each of the arrows shows the direction in which a particular “-omic” influences another. In the case of fluxomics, it combines all approaches, granting better understanding. Dauner describes observed flux/activity as a two component - capacity-based and kinetics-based - regulation (Figure 3). Created with Biorender.com.

Fluxomics is a new metabolomics application, which is focused on actual rates within metabolic networks. Since the reaction rates (fluxes) of metabolic pathways cannot be measured directly due to the intrinsic properties of metabolism such as dynamics, the fluxes can be measured indirectly by the shifts in metabolite levels (Cascante and Marin, 2008; Winter and Krömer, 2013). What distinguishes fluxomics from other–omics is the fact that the fluxome (total set of fluxes in metabolic network of a cell) occur as a resultant of all other “–omes” combined (mainly the proteome and the metabolome). While the genome, transcriptome, proteome and metabolome focus only on their own elements–for example the interactions between proteins in the proteome–the fluxome captures the real and dynamic picture of phenotypes by observing the interactions between all of the “-omes”, therefore granting a unique synergistic insight (Cascante and Marin, 2008; Aon and Cortassa, 2015) (Figure 2).

**FIGURE 3:**
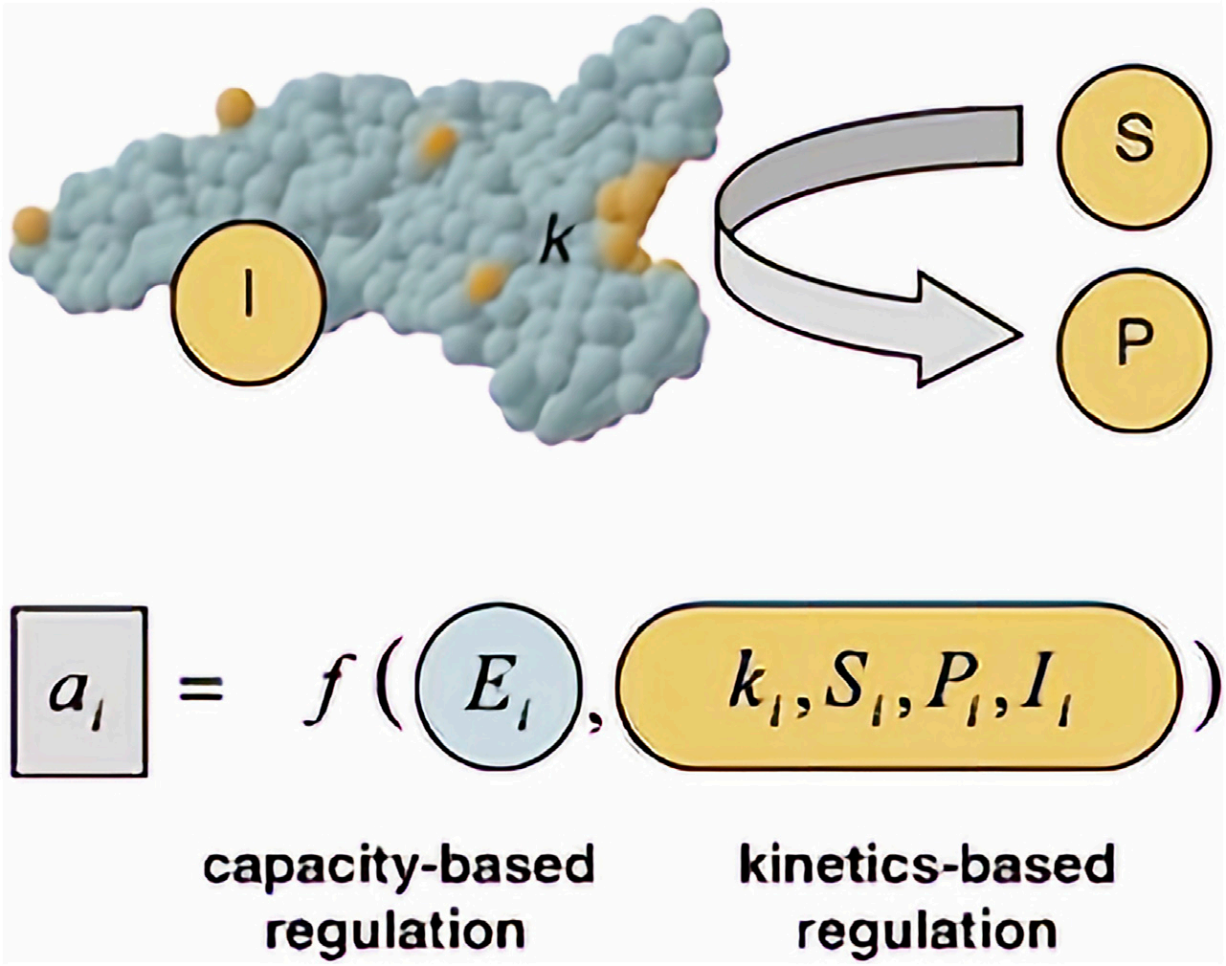
Observed flux/activity *a* of a reaction step I. Adapted with permission from (Dauner, 2010).

Capacity-based regulation is a function related strictly to gene regulatory processes of the cell. Those processes such as enzyme production and stability within the cell (E_i_) will differ, depending on the cell and its function in a multicellular organism. As for kinetic regulation, it is a function of kinetic parameters of enzymes catalyzing the reaction (k) (accounting also for enzyme modifications such as phosphorylation), concentration of substrate (S) and product (P) and effector/signaling molecules (I). (Dauner, 2010). Those variables can be measured by using e.g. quantitative proteomics to calculate enzyme concentration (E_i_) (Ong et al., 2003; Mann, 2006; Winter and Krömer, 2013) and quantitative metabolomics for substrate, product and effector concentrations (Winter and Krömer, 2013).

**FIGURE 4:**
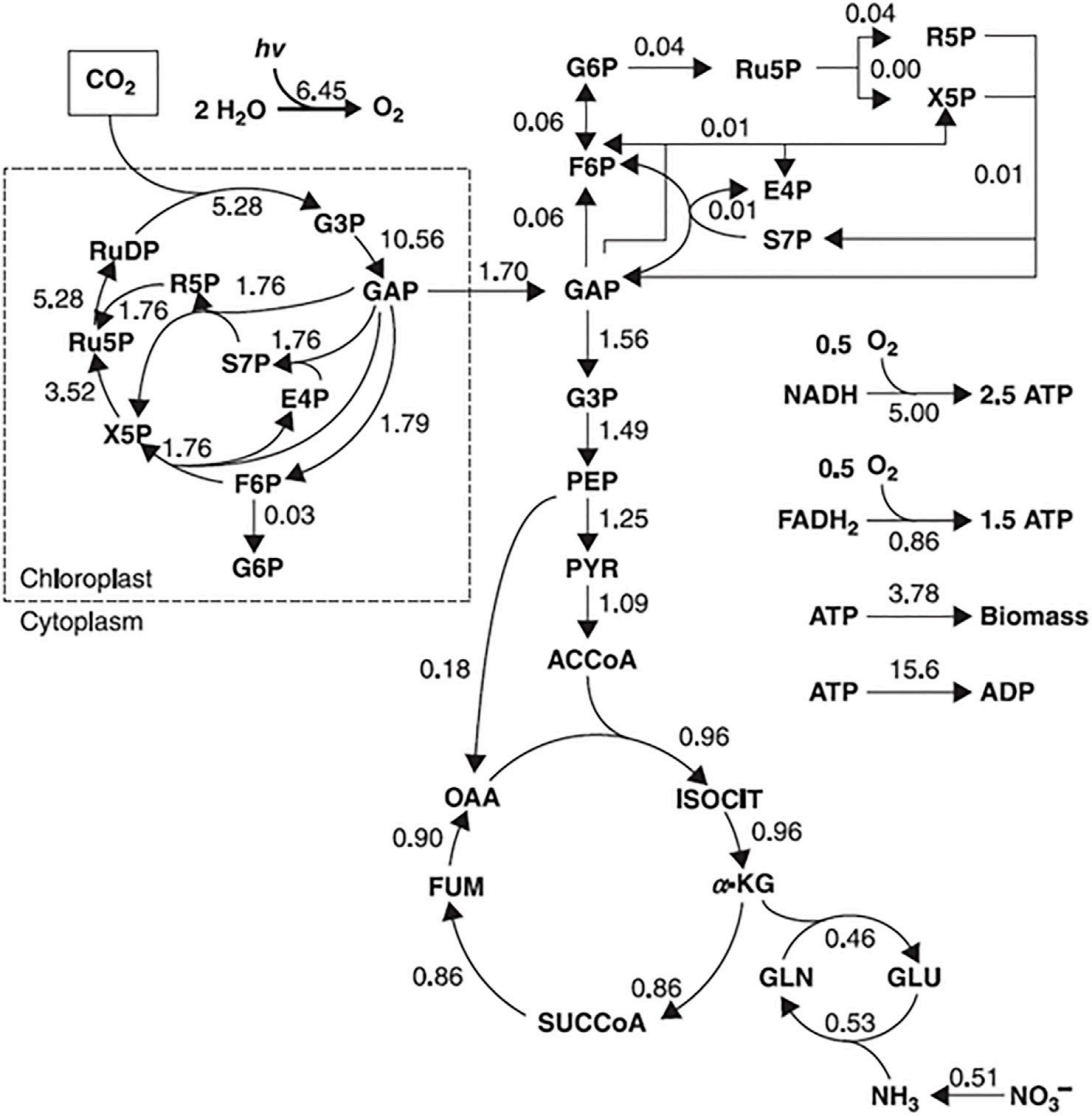
Example of a flux map, representing a metabolic flux distribution of Chlorella cells in autotrophic cultures. The flux values are expressed in mmol/g/h. Adapted with permission from (Shimizu and Shimizu, 2013).

The type of approach used to describe the metabolic network will depend on its nature. For example, metabolic flux analysis (MFA) identifies the whole set of fluxes in a part of the metabolic network of a microorganism *in vivo* (Wiechert et al., 2005). Information about fluxes is obtained by assuming an intracellular pseudo-steady state (a state, where intracellular metabolites do not accumulate in the cell and the balance between the consumption and production fluxes of a metabolite is in equilibrium) and reaction stoichiometry (a fixed configuration of the metabolic network that does not account for cell adaptation to the environmental changes), to estimate the balances around intracellular metabolites, by calculating the uptake rates of substrates and secretion rates of metabolites (Stephanopoulos et al., 1998; Provost and Bastin, 2006; Antoniewicz, 2015). Those rates are measured by monitoring external rate changes such as substrate consumption (glucose uptake rate), biomass synthesis (growth rate), energy consumption and production (CO_2_ evolution rate), and metabolite production. The final result is a metabolic flux map with an estimate of the flux of each reaction (Figure 4).

For mathematical explanation of the flux calculation, the reader is referred to (Stephanopoulos et al., 1998; Provost and Bastin, 2006; Shimizu and Shimizu, 2013). A variant of MFA called dynamic metabolic flux analysis (DMFA) focuses on describing metabolic fluxes in a metabolic non-steady state, in which a time-series of extracellular concentration and rate measurements are used. In this approach the experiment is divided into a set of time intervals from which the external rates are calculated for each time interval. Then the results are averaged and combined to obtain a time profile of related fluxes (Antoniewicz, 2013; Antoniewicz, 2015).

**FIGURE 5:**
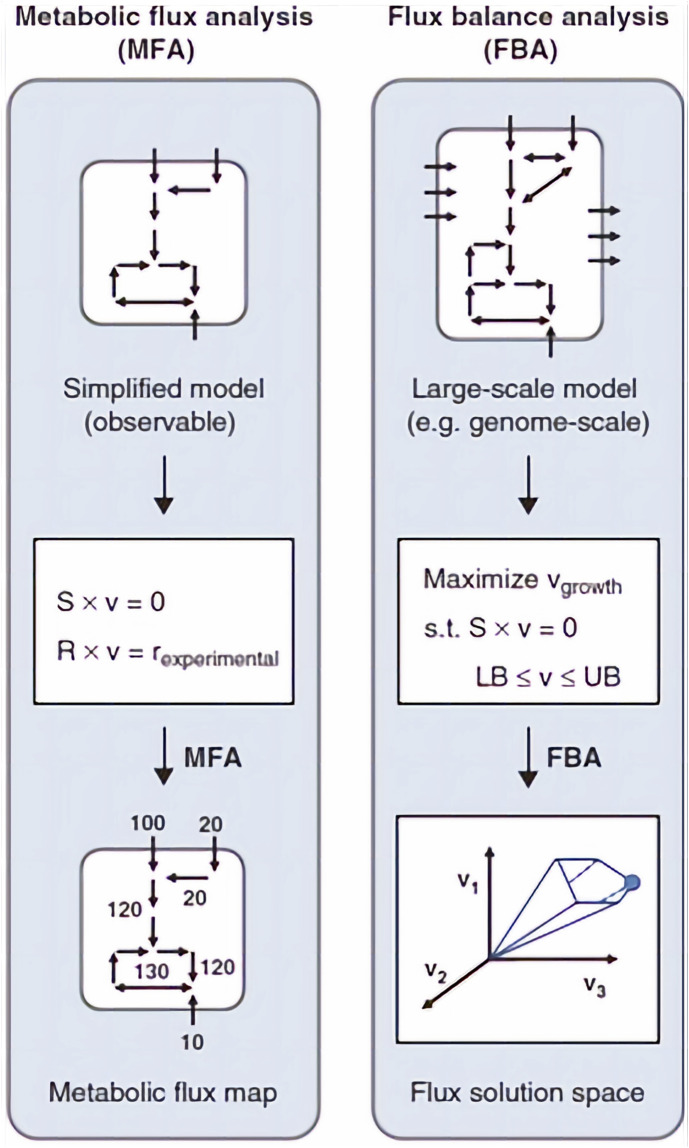
The basics of MFA and FBA approaches. *S* is the stoichiometric matrix, *v* is the flux vector, r is the external metabolic rates. In MFA, fluxes are calculated by fitting extracellular rates measured experimentally. In FBA, a flux solution space is determined by assuming a biological objective, for example, maximization of growth rate, and solving a linear optimization problem. Adapted with permission from (Antoniewicz, 2015).

Another approach to describe a metabolic network is called flux balance analysis (FBA). When compared to MFA, FBA works on a broader scale, and it enables reconstruction of a metabolic network on the genome-scale level. These reconstructions utilize all information about metabolic reactions in an organism and the genes that encode each enzyme. However, this approach does not count for regulatory interactions and detailed kinetics, giving only partial biological information of the situation of systems at steady state. To obtain fluxes from FBA, first a reconstructed metabolic network must be converted to a mathematical matrix. Within this matrix, a set of constraints is imposed by mass balance equations and reaction bounds. Then, based on the biological objective (e.g., biomass production), linear programming is used to determine the sought fluxes by either maximizing or minimizing objective function while considering given constrains (Orth et al., 2010; Antoniewicz, 2015; Aon and Cortassa, 2015). The differences between the MFA and FBA approaches are shown in Figure 5.

Extracellular fluxes between different cells and their environment can also be determined by using ^13^C-isotope substrate followed by NMR monitoring of ^13^C-labeled metabolite propagation through the metabolic intermediates in certain metabolic pathways. ^13^C-labeled fluxomics is an extension of FBA in which all the precursors (substrates) used by the cells are ^13^C-enriched. Consumed substrates are later incorporated into the metabolic pathways connected to the used substrate. The level of incorporation will depend on intracellular fluxes that could be measured using NMR and/or MS. The information obtained from those experiments can be utilized to discriminate metabolic variants (isotopic profiling), measure specific fluxes (targeted flux analysis–TFA) and investigate the whole fluxome (global fluxomics) (Wiechert et al., 2001; Krömer et al., 2008; Heux et al., 2017). For example, GC-MS and NMR were employed to monitor metabolic flux in neural stem cells (NSCs) using labeled carbon -^13^C glucose. By following ^13^C labeling pattern and monitoring an isotopic non-stationary metabolic flux analysis, it was demonstrated that pyruvate entered the tricarboxylic acid (TCA) cycle mostly through pyruvate carboxylase (81%) (Sá et al., 2017). Another practical example of isotope labelling is to identify isotopomers (one of the different labeling states in which a particular metabolite can be encountered). The isotopomer redistribution of a metabolite is calculated based on the percentage value of each isotopomer within the metabolite pool. The information obtained from such an approach describes how the various isotopomers react with each other (Wiechert et al., 2001). Isotopic labelling is not limited only to ^13^C. Other elements such as ^15^N, ^18^O or ^31^P can also be used to study, e.g. nitrogen metabolism and muscle energetics (Klein and Heinzle, 2012; Nemutlu, 2015).

Isotope labelling was recently used to determine whether pyruvate or glutamine are anaplerotic sources requiring pyruvate carboxylase (PC) and glutaminase 1 (GLS1) activity. Sellers et al. (Sellers et al., 2015) utilized NMR-based metabolomics approaches to monitor the Krebs cycle of patients with early-stage non–small-cell lung cancer (NSCLC) infused with uniformly ^13^C-labeled glucose followed by tissue resection. NMR analysis of patient cancerous tissues showed enhancement of pyruvate carboxylase (PC) activity. Furthermore, results from patient cancer tissues cultured in ^13^C_6_-glucose or ^13^C_5_,^15^N_2_-glutamine tracers provided clear evidence of selective activation of PC over glutaminase (GLS) in NSCLC (Sellers et al., 2015).

Another prominent example of isotope labelling used for fluxomics is work by Cocuron *et. al.* (Cocuron et al., 2019), comparing the metabolism of two different maize lines - Alex and LH59. The goal of this work was to test if a change in carbon metabolism may increase oil content in maize kernels to help sustain the demand for vegetable oil. Cocuron et al. labeled Alex embryos with ^13^C labeled glucose and utilized NMR, GC-MS and LC-MS/MS to measure carbon flow through the metabolic network (^13^C-MFA). Alex line embryos (which accumulate more oil when compared to LH59) increased the amount of Glucose 6-phosphate (G6P) entering into the plastid, the aldolase in the plastid, the export of TPs (Glyceraldehyde 3-phosphate) to the cytosol, the glycolytic flux in the cytosol, Phosphoenolpyruvate carboxylase (PEPC), and plastidic malic enzyme. It was concluded that increasing the levels of plastidic malic enzyme should enhance the fatty acid content of seeds (Cocuron et al., 2019).

In the recent studies, *Bergès et al.* utilized both NMR and MS approaches to obtain high resolution fluxotypes for huge numbers of a strains in a library. They define fluxotype as “*the particular distribution of metabolic fluxes measured for a given strain under given physiological conditions*” (Bergès et al., 2021). The authors studied the fluxotype of 180 different *E. coli* strains with deleted y-genes. Bacteria were grown in ^13^C labeled glucose as a single source of carbon while monitoring metabolic fluxes. Deletion of two y-genes led to a significant modification of metabolic fluxes indicating the role of the studied genes in metabolic regulation (Bergès et al., 2021).

Both NMR and MS have been frequently used to investigate the impact of fluxomics in drug delivery and pharmacology. For instance, the production of artemisinic acid in an engineered *E. coli* strain that encodes *S. cerevisiae* enzymes allows the cell to enter the mevalonate pathway and supplement endogenous isopentenyl pyrophosphate (IPP) biosynthesis. This then enhances the production of the antimalarial drug artemisinin. This shift in pathways relies on the flux rate and metabolites concentration (Ro et al., 2006).

In addition, the emergence of multi-drug resistant strains of *tuberculosis* provides a need to develop additional medications for disease treatment. The application of fluxomics to target metabolic enzymes and genome-scale models can be used for analysis, discovery, and as hypothesis-generating tools, which will hopefully assist the rational drug development process. These models need to be able to assimilate data from large datasets and analyze them. A study in 2007 reconstructed the metabolic network of *Mycobacterium tuberculosis* H37Rv (Jamshidi and Palsson, 2007). This strain can produce many of the complex compound’s characteristic to *tuberculosis*, such as mycolic acids and mycocerosates. Researchers in this study grew this bacterium *in silico* on various media, analyzed the model in the context of multiple high-throughput data sets, and finally they analyzed the network in an ‘unbiased’ manner by calculating Hard Coupled Reaction (HCR) sets and FBA. The results showed growth rates comparable to experimental observations in different media, and by considering HCR sets in the context of known drug targets for *tuberculosis* treatment they proposed new alternative, but equivalent drug targets (Jamshidi and Palsson, 2007).

**FIGURE 6:**
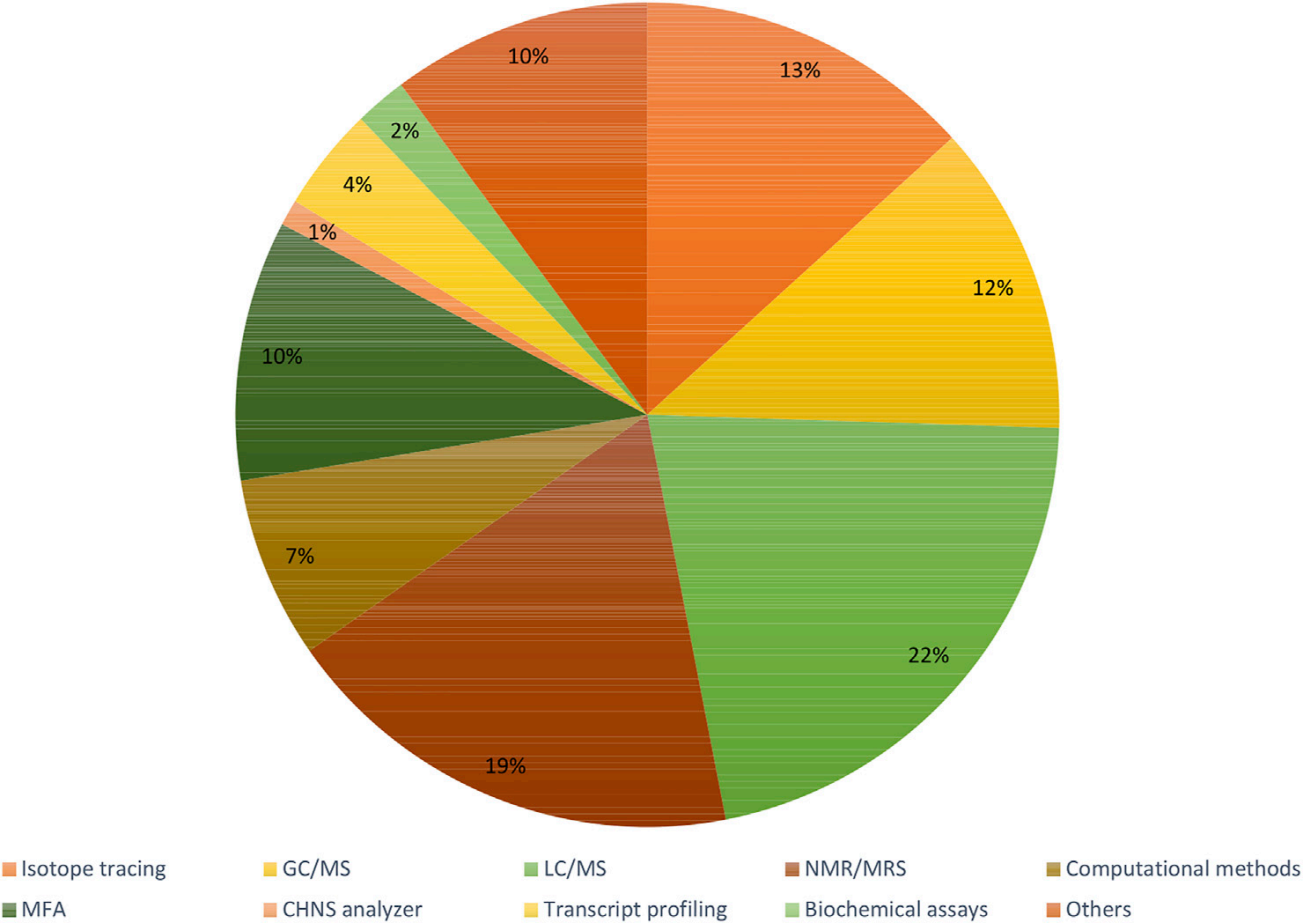
Summary of the most used techniques within fluxomic studies.

**FIGURE 7:**
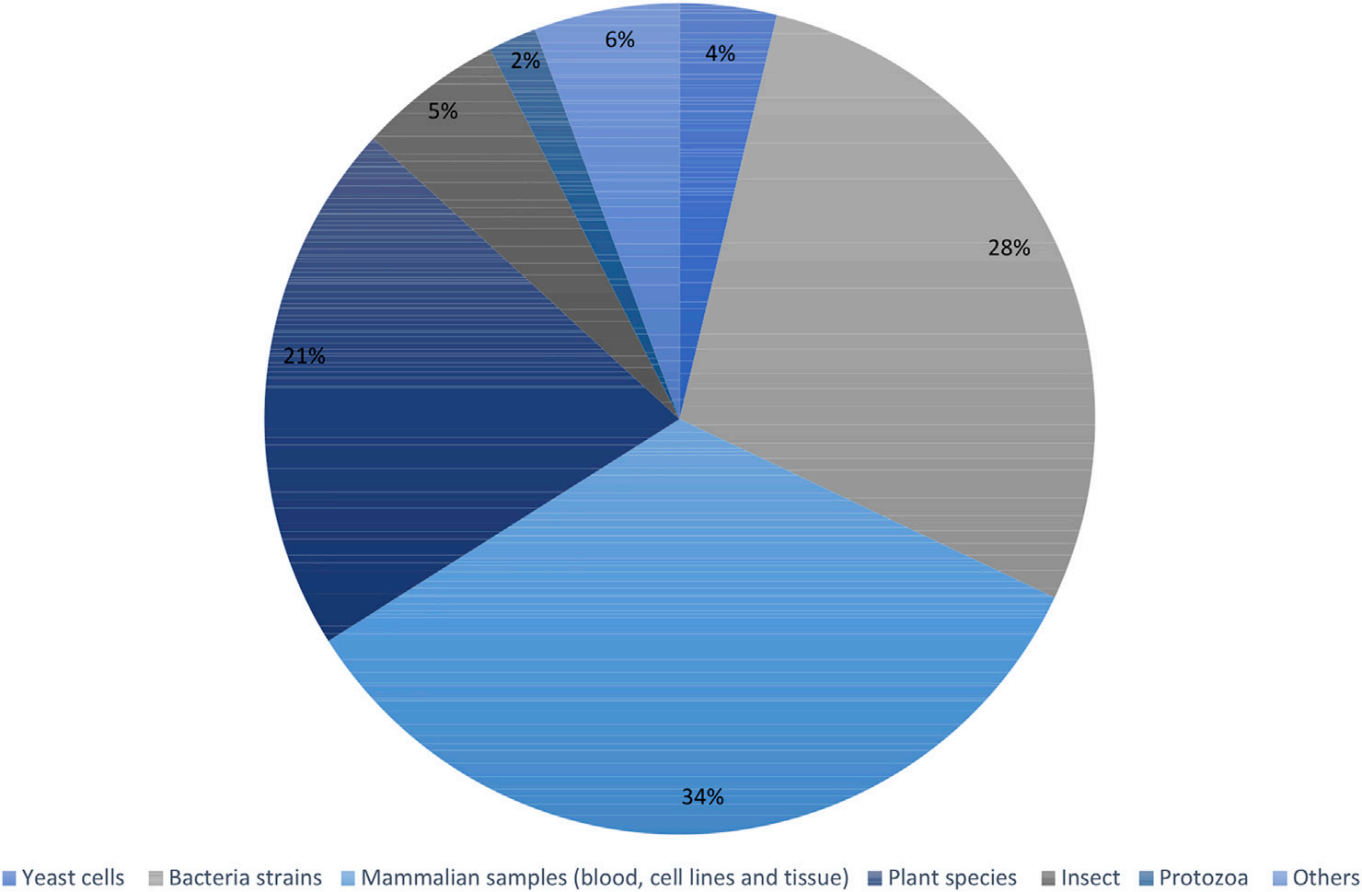
Summary of the most used organisms within fluxomic studies.

**FIGURE 8:**
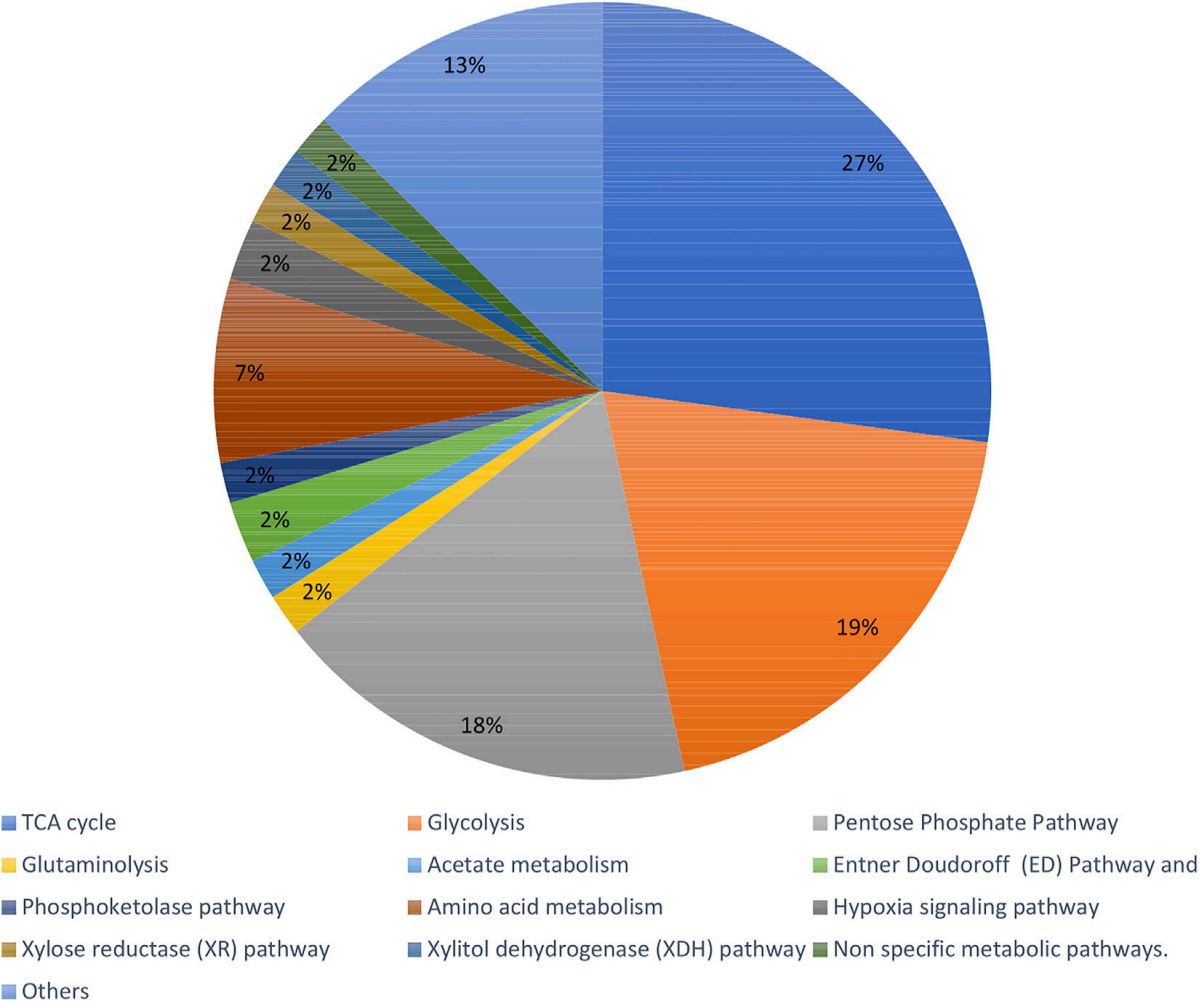
Summary of the commonly described pathways within fluxomic studies.

Recent articles proving the constant increase in popularity of the fluxomic field have been collected in Supplementary Table S1. The summary of most popular techniques, organisms and pathways described within the studies are shown in Figures 6–8.

## Fluxomics Databases

Rising interest in–omics fields (i.e., proteomics, genomics, and metabolomics) has resulted in an increased number of recent studies. The massive amount of data produced from these studies must be properly managed to increase its accessibility. This has given rise to various -omics databases such as PeptideAtlas (http://www.peptideatlas.org/) (Deutsch et al., 2008), PRIDE (https://www.ebi.ac.uk/pride/) (Martens et al., 2005) (proteomics related databases), Human Metabolome database (HMDB) (www.hmdb.ca) (Wishart et al., 2007) and METLIN (https://metlin.scripps.edu/) (Smith et al., 2005) (metabolomics related databases).

Fluxomics still has not reached its true potential, partly due to a lack of uniform data standards in the reconstruction of metabolic networks (Crown and Antoniewicz, 2013) (Thiele and Palsson, 2010). Therefore, there is an emerging need to construct new and user-friendly databases, which not only store, but also match flux results and create metabolic network models. Recently, novel solutions to reach these ambitious goals have been developed and are briefly described here.

The Central Carbon Metabolic Flux database (CeCaFDB, available at http://www.cecafdb.org) is a novel database published in 2014 that focus on central carbon metabolic systems of microbes and animal cells. The database contains 581 cases of quantitative flux results among 36 organisms including: *Homo sapiens*, *Escherichia coli*, *Saccharomyces cerevisiae* and *Pichia pastoris*. Based on user input it can utilize four modules (vector-based similarity, a stoichiometry-based comparison, a topology-based similarity, and enzyme-topology based similarity) for comparison and alignment of different flux distributions. Additionally, this database provides the opportunity to perform similarity calculations by utilizing deposited data and altering genetic and environmental factors (Zhang et al., 2015).

Datanator (https://datanator.info) is an integrated multisource database that contains quantitative molecular data of several types including metabolite concentrations, RNA modifications and half-lives, protein abundances and modifications, and reaction rate parameters. Developed in 2020, Datanator includes various data for 1,030 organisms integrated from over 8,000 articles. Although it does not contain flux related data yet, the authors are planning to include it in the near future, as well as information on RNA/protein localizations and protein half-lives. In such case, Datanator would be a valuable source for comparative analyses of relationships between variable networks and systems (Roth, 2021).

BiGG Models (http://bigg.ucsd.edu) is a large-scale database containing genome-scale metabolic network reconstructions. It contains more than 100 genome-scale metabolic models. Those models contain information about biochemical reactions, metabolites and genes related to the metabolism of specific organisms. The information provided in BIGG Models is standardized across different models, which allows users to browse, share and visualize the networks in a structured manner (King, 2015).

Besides those three databases, various other databases used in different–omics fields can be used to obtain partial information that can be useful for studying the fluxes. Some of them are listed in Table 1.

**TABLE 1:** Examples of databases useful for fluxomic-related studies.

Database	Link	Brief description	Ref
Central Carbon Metabolic Flux database (CeCaFDB)	www.cecafdb.org	Contains 581 cases of quantitative flux results among 36 organisms. CeCaFDB can be used for comparison and alignment of different fluxes and to understand how they are changed by other factors	Zhang et al. (2015)
Datanator	www.datanator.info	Multisource database containing information about metabolites, RNA, proteins and reactions. Datanor will include information about fluxes in near future, in which case it could be used for comparative analyses of relationships between variable systems and their constituents	Roth, (2021)
BiGG Models	www.bigg.ucsd.edu	Contains more than 100 genome-scale metabolic network reconstructions that provide information about biochemical reactions, metabolites and genes related to metabolism for a specific organism	King, (2015)
The Human Metabolome database (HMDB)	www.hmdb.ca	Contains 220,945 metabolite entries (both water-soluble and lipid soluble) with 8,610 protein sequences (enzymes/transporters) linked to them including pathways and reactions related to the metabolite. Provides users with data obtained by MS and NMR analyses performed on urine, blood, and cerebrospinal fluid samples	(Wishart et al., 2007; Wishart et al., 2018)
SABIO-RK	www.sabio.h-its.org	Contains information about biochemical reactions and their kinetics. Provides the user with information about the involvement of reaction in various pathways, modifiers of reaction enzymes involved in reactions and measured kinetic data (including kinetic rate equations)	Wittig, (2011)
Braunschweig Enzyme database (BRENDA)	www.brenda-enzymes.org	The largest depository of all classified enzymes, including biochemical and molecular information. The database includes information such as enzyme class, reaction in which the enzyme is involved, specificity of reaction, functional parameters of the reaction, localization of enzyme, the application of enzymes, and ligand-related data	Chang et al. (2009)

## Conclusion

Matching genomic, transcriptomic, proteomic, and metabolomic data is essential for global understanding of biological systems. Fluxomics provides insight into actual rates within metabolic networks, both because of both cellular activity and environmental changes. Such knowledge can be obtained using different approaches including metabolic flux analysis (MFA), dynamic metabolic flux analysis (DMFA), flux balance analysis (FBA) or ^13^C-labeled metabolite monitoring. In addition to this wide variety of approaches in fluxomics, the significant advances in instrumentation methods such as NMR and MS, along with new databases and software, increase the prevalence of fluxomics studies. Nowadays, fluxomics gives rewarding data of complexed multi-molecular interactions in biological systems, which has never been observed before.
