# Supplementary material for: Fluxomics - New Metabolomics Approaches to Monitor Metabolic Pathways
Source: Front Pharmacol. 2022 Mar 21;13:805782. doi: 10.3389/fphar.2022.805782 (PMC8977530; doi:10.3389/fphar.2022.805782)
Supplement: Supplementary file 1 [file Table1.docx]

**Supplementary Table 1.** Examples of recent studies employing fluxomics

| **Goal** | **Experiment** | | | **Method** | | | **Ref** |
| --- | --- | --- | --- | --- | --- | --- | --- |
|  | **Model** | **No. of samples** | **Target omics** | **Platform** | **Type of Analytes** | **Pathways** |  |
| GC/Q-TOF MS with a novel low-energy source to optimize metabolites and deliver sufficient relative and absolute signal intensity of the selective fragment [M − CH3]^•+^ | Yeast cells | 17 | ^13^C-based metabolic flux analysis | GC-​QTOF-MS | Organic acids, redox factors, nucleotide related, coenzyme A, sugar phosphates | Non specific pathway | (1) |
| Detailed maps of carbon fluxes in central carbon metabolism, produced in a fully automated manner. This was used to investigate the glucose fluxotypes of 180 *E. coli* strains deleted for y-genes | *E.coli* | 180 | High-resolution fluxotyping; y-ome phenotyping.  ^13^C-based metabolic and isotopes | NMR /MS | Sugar phosphate, amino acids, organic acids, redox factors, nucleotide related, coenzyme A and salts | NADPH production, Pentose-Phosphate Pathway (PPP),  TCA cycle, Oxidative metabolism, FADH_2_  production, glycolysis, ATP production, acetate metabolism | (2) |
| Kinetic isotope labeling experiments, LC-MS, and computational analyses that relate kinetic isotope trajectories of metabolites to pathway activity | Cyanobacteria | N/A | Isotopic substrates:  sodium ^13^C-bicarbonate/^13^CO2,  U-^13^C-glutamate | Kinetic isotope labeling experiments, (LC-MS), and computational analysis | Sugar phosphate, fatty acids, organic acids, salts, redox factors | TCA cycle, fatty acid synthesis, glycolysis, pentose phosphate pathway and non-mevalonate pathway | (3) |
| Temporal-fluxomics to derive a comprehensive and quantitative view of alterations in metabolic fluxes throughout the mammalian cell cycle. This was achieved by combining pulse-chase LC-MS-based isotope tracing in synchronized cell populations with computational deconvolution and metabolic flux modeling | Hela cells | 3 | Labelled [U-^13^C]-glutamine | Pulse-chase LC-MS-based isotope tracing | Organic acids, salts, redox factors, redox factors, nucleotide related and amino acids | TCA cycle | (4) |
| Exploration of the ALSOFAST-HSQC experiment with its rapid acquisition scheme for the analysis of ^13^C-labeled metabolites in complex biological mixtures. As an initial step, the parameters of the pulse sequence were optimized to account for characteristics of complex samples. A fast two-dimensional experiment was used to study the effects of different kinds of antioxidant gold nanoparticles on a HeLa cancer cell grown in ^13^C glucose-enriched medium | Hela cells | 3 | Labelled ^13^C glucose | Fast NMR/ ^1^H,^13^C-HSQC | Organic acids, salts, redox factors, amino acids and sugar phosphate | Pentose phosphate pathway and TCA | (5) |
| Fluxomics combined with isotopic non-stationary metabolic flux analysis (^13^C-MFA) to quantify the metabolic rewiring accompanying astrocytic lineage specification from neural stem cells | The murine embryonic stem cell (mESC) line CGR8 | 3 | [1-^13^C] glucose  Tracer | ^1^H-NMR; GC-MS | Sugar phosphate, amino acids, organic acids, redox factors, nucleotide related, coenzyme A and salts | Glycolysis, pentose phosphate pathway, TCA cycle and amino acids metabolism | (6) |
| Microsampling with hemaPEN^®^ followed by targeted metabolomics to follow-up metabolic profiles. This study used two UHPLC-MS/MS methods to quantify 13 metabolites. The results open new avenues for fluxomics in health care | Female and male blood samples | 20 | Microsampling with hema PEN®  followed by targeted metabolomics | UHPLC-MS/MS | Amino acids, organic acids, redox factors | TCA cycle and amino acids metabolism | (7) |
| Understanding amino acid metabolism by demonstrating a link between amino acid changes and IgG production. Illustrated by a genome-scale model and validated by transcriptomics analysis. A reference computational tool both for basic process understanding and for in silico simulation of cell culture conditions | Chinese hamster ovary (CHO) cells | 4 | *In silico* simulation | Genome-scale CHO model | Sugar phosphate, amino acids, organic acids, redox factors, nucleotide related, coenzyme A and salts | N-glycan biosynthesis, glycolysis/gluconeogenesis, valine, leucine and isoleucine degradation, TCA cycle, alanine metabolism, amino sugar and nucleotide sugar metabolism | (8) |
| Measuring transcriptomic, proteomic, phospho-proteomic and fluxomics data in a breast cancer cell-line (MCF7). Integrating these multiomics data within a genome scale human metabolic model in combination with machine learning, predicting which enzymes and pathways are regulated at which level | MCF7 cells | 3 | Integration of data within a genome scale metabolic model (GSMM) of human metabolism. ^13^C labeling | GCMS-QP 2; GC-MS; (13C MFA) Metabolic flux analysis; Genome scale human metabolic model | Sugar phosphate, amino acids, organic acids, redox factors, nucleotide related, coenzyme A and salts | Glycolysis, lactate production, pentose phosphate pathway (PPP) activity, tricarboxylic acid cycle (TCA) cycle utilization and fatty acid synthesis | (9) |
| Comparative metabolomics and fluxomics to enhance fatty acid synthesis (FAS) in maize (*Zea mays*), comparing two lines. Comparative metabolomics highlighted the metabolites and pathways that were active in the embryos and important for oil production. This approach combined mathematical modeling with biochemical quantification to identify metabolic bottlenecks in FAS in maize embryos | Maize embryos from two different maize lines, Alex and LH59 | 6 | ^13^C-labeling  [U-^13^C6]-Glc, [U-^13^C6]-Fru, and [U-^13^C5]-Gln isotopes | ^13^C-metabolic flux analysis | Sugar phosphate, organic acids, redox factors, coenzyme A and salts | Glycolysis and pentose phosphate pathway (PPP) activity | (10) |
| A combined fluxomic and metabolomic approach to study sperm metabolism in felids, demonstrating a proof of concept that significant differences in metabolic output can be detected using small sample volumes. These findings hold potential not only for further studies on sperm metabolism, but also for oocytes and embryos whose small sizes have posed limitations on metabolic studies using GC-MS | Spermatozoa | 97 | ^13^C-labelled substrates | GC-MS | Sugar phosphate, amino acids, organic acids, redox factors, nucleotide related, coenzyme A and salts | Glycolysis and/or oxidative phosphorylation pathway for ATP production | (11) |
| Investigating central carbon metabolism fluxes and central cell bioenergetics in response to ammonium availability and nitrogenase activity. This is done by ^13^C-metabolic flux analysis. This study provides a quantitative relationship between central carbon and nitrogen metabolism in an aerobic diazotroph for the first time | *Azotobacter vinelandii* DJ (wt) and AV3 (ΔNifL) | 3 | ^13^C-metabolic flux | GC-MS measurement for labeled amino acids; Residual glucose, ammonium, PHB and Alginate quantification; ^13^C-metabolic flux analysis | Sugar phosphate, organic acids, redox factors, nucleotide related, coenzyme A and salts | TCA, Entner Doudoroff  (ED) Pathway and  oxidative pentose phosphate (OPP) pathway | (12) |
| Advanced metabolomic/fluxomic techniques for comprehensive, precise, and prompt evaluations of the effects of kinase inhibition in MCL cells. This has strong translational implications by potentially permitting early evaluation of cancer patient response versus resistance to kinase inhibitors and in design of novel therapies against disease resistance | MCL-RL cells | 3 | [U-^13^C_5_, U-^15^N_2_] glutamine | LC/MS; ^1^H MRS; ^13^C MRS | Sugar phosphate, amino acids, organic acids, redox factors, nucleotide related, coenzyme A and salts | Glycolysis, TCA cycle, glutaminolysis, and PPP | (13) |
| Applying (1) H-nuclear magnetic resonance ((1) H-NMR) to analyze the supernatants of glutamine-synthetase (GS)-CHO cell clones expressing variable amounts of an IgG4 under control and butyrate-treated conditions. Exometabolomic data revealed accumulation of several metabolic by-products, indicating inefficiencies at different metabolic nodes | CHO cells | 3 | N/A | ^1^H-NMR | Sugar phosphate, amino acids, organic acids, redox factors, nucleotide related, coenzyme A and salts | Glycolysis, PPP, TCA cycle, glutaminolysis, amino acids metabolism, by-product formation and extracellular transport fluxes | (14) |
| Spatial fluxomics to infer metabolic fluxes in mitochondria and the cytosol under physiological conditions, combining isotope tracing, rapid subcellular fractionation, LC-MS-based metabolomics, computational deconvolution, and metabolic network modeling. Applied to the study of reductive glutamine metabolism in cancer cells, shown to mediate fatty acid biosynthesis under hypoxia and defective mitochondria. This spatial-fluxomics approach is a useful tool for elucidating the role of metabolic dysfunction in human disease | HeLa cells | 3 | Isotopic [U-^13^C]-glucose or [U-^13^C]-glutamine | Mitochondria isolation and isotope tracing; LC-MS analysis; Compartmentalized metabolic flux analysis (MFA) | Fatty acids, amino acids, coenzyme A and salts and Sugar phosphate | TCA cycle | (15) |
| Using stable isotope tracer to explore metabolic kinetics in mice in vivo and in vitro. This determines systemic flux rates, and intracellular metabolic pathway flux rates (i.e., relative flux rates) through the close examination of MID patterns. The absolute intracellular flux rates of a specific metabolic network can also be estimated with the assistance of computer software such as INCA | Mice (in vivo and in vitro) | 6 | Labeled 1-^13^C] glucose) , ([U^13^C_6_]  glucose; [U]) or labeled [6,6-^2^H_2_]glucose) | *In vivo*: Metabolic flux analysis (MFA); *in vitro*: Mass isotopomer distribution (MID); GC-MS and external flux rates with tracer | Amino acids, sugar phosphate, organic acids, redox factors, nucleotide related, coenzyme A and salts | Glycolysis, TCA cycle, and oxidation | (16) |
| A validated computational model of heart central catabolism, comprising glucose and fatty acid (FA) oxidation in cytoplasmic and mitochondrial compartments by estimating fluxes through glucose degradation pathways. | Mice (myocytes) | 2 | Untargeted metabolomics | Bioinformatic analyses MetaboAnalyst 3.0; Computational modelling of central catabolism; Kinetic Modelling of metabolic networks; MATLAB | Sugar phosphate, amino acids, organic acids, redox factors, nucleotide related, coenzyme A and salts | Polyol, pentose phosphate, TCA cycle, β-oxidation, on the redox status (NADPH, ROS) | (17) |
| Bioinformatic analysis integrated with metabolic, transcriptomic, fluxomic and mutant data into a multi-level kinetic model. This model helps in solving problems linked to neglected isozyme, *pket*2 (*sll0529*). Results imply that the PKET pathway plays a crucial role under AC-auto by mitigating the decarboxylation occurring in OPP pathway and the conversion of pyruvate to acetyl CoA linked to EMP glycolysis under the carbon scarce environment. | [Cyanobacteria](http://en.wikipedia.org/wiki/Cyanobacteria) (*Synechocystis* sp) | 2 | ^13^C labeling data | ^13^C labeling data; multi-level kinetic model | PKET, Sugar phosphate, amino acids, organic acids, redox factors, nucleotide related. | Phosphoketolase  pathway | (18) |
| Understanding the metabolic dependence of *Wolbachia* on its host, the possibility of metabolic provisioning and extraction, and the interplay with available dietary iron, using NMR-based metabolomics and metabolite profiles of *Wolbachia*-infected and uninfected *Drosophila melanogaster* flies raised on varying levels of dietary iron. The metabolic response to infection showed a reprogramming of the mitochondrial metabolism in the host. Based on these observations, and a physiological model which postulates that the host's insulin/insulin-like-growth factor pathway is depressed and the hypoxia signaling pathway is activated upon *Wolbachia* infection | *Drosophila melanogaster* | 6 | Target genes amplified using SYBR-Green pre-mix | NMR | Aminoacids, sugars, lipids, and organic acids | Hypoxia signaling pathway | (19) |
| Determining flux by the activity of individual cell components. By pulse labelling of the organism with a tracer such as ^13^C, followed by mass spectrometric analysis of the partitioning of label into different compounds. The use of flux sensors, proteins that respond with a conformational change to ligand binding | *Arabidopsis* root | N/A | ^13^C-fluxomics | ^13^C-based flux analysis; MS | Amino acids, sugars, salts and other FRETs | Signaling pathway | (20) |
| Investigating the reliability of 2D-COSY and 2D-TOCSY experiments to provide accurate measurements of ^13^C-enrichments in complex mixtures of ^13^C-labelled metabolites. The results showed that 2D-TOCSY but not 2D-COSY could provide accurate measurements of ^13^C-enrichments, provided efficient zero-quantum filters were applied during the mixing period. This approach extends the range of NMR methods applicable in ^13^C-labelling experiments and is suitable to the investigation of the dynamic behavior of metabolic systems | *E. coli* | 10 | [1-^13^C]-glucose | NMR (2D-TOCSY, 2D-COSY) | Glucose, amino acids (Leu and Val) | Metabolic network | (21) |
| Development of isotope tracing, mass spectrometry, and mathematical analysis methods to determine the direct sources of circulating nutrients, their interconversion rates, and eventual tissue-specific contributions to TCA cycle metabolism. The presented *in vivo* flux quantification methods are broadly applicable to different physiological and disease states | Mice | N/A | 15 different ^13^C-isotope tracers | LC-MS | Circulating metabolites, fatty acids, amino acids and coenzyme A and salts | TCA cycle | (22) |
| Since changes in the metabolic network reflect interactions between genetic, epigenetic and environmental changes, it is helpful to study the flow of label from isotopically labelled precursors into other metabolites rather than static metabolite levels. NMR is an attractive technique for this as it can quantify site-specific label incorporation. However, for applications using human cells, the challenge is to optimize the process to maximize sensitivity and reproducibility. This is represented by a new framework to analyze metabolism in mammalian cell lines and primary cells, covering the workflow from the preparation of cells to the acquisition and analysis of NMR spectra. This new approach is shown to be feasible in hematological and liver cancer cell lines | Hematological and liver cancer cell lines | N/A | Unlabelled and ^13^C or ^15^N- labelled precursors. | D-^1^H-NOESY and a 2D-^1^H, ^13^C-HSQC,  2D-^1^H,^1^H-TOCSY spectra (NMR), MS | Sugar phosphate, amino acids, organic acids, redox factors, nucleotide related, coenzyme A and salts | Glycolysis, the oxidative and non-oxidative branch pentose phosphate pathway (PPP), Krebs cycle metabolism, the relative importance of pyruvate dehydrogenase and pyruvate carboxylase, TCA cycle | (23) |
| Metabolic flux modeling is a complementary technique to standard metabolomics as routinely practiced in biological systems. Carbon-13-based isotopomer analysis using nuclear magnetic resonance as the primary analytical technique. Isotopomer analysis produces relative rates of tricarboxylic acid cycle turnover. If these measures are normalized to O_2_ consumption, absolute rates can be inferred. The primary biological system in this review is cell culture | Cancer cell lines | N/A | ^13^C labeled isotopes. | ^13^C and ^1^H NMR | Sugar phosphate, amino acids, organic acids, redox factors, nucleotide related, fatty acids and coenzyme A and salts | TCA cycle | (24) |
| Measuring fluxomics by NMR and incorporation of isotope labels in selected metabolites to follow kinetically the synthesis of metabolites. Investigating different NMR approaches to optimize such experiments in terms of resolution and time requirement. Using isoleucine biosynthesis as an example | *E. coli* K-12 MG1655 | 3 | ^13^C-labeled glucose and unlabeled | NMR | Amino acids and sugar phosphate | Biosynthetic pathways for Leu/Val and Ile synthesis | (25) |
| Demonstrating the ability to directly track, by liquid chromatography–mass spectrometry, the passage of deuterium from labelled substrates into NADPH, and combining this approach with carbon labelling and mathematical modelling to measure NADPH fluxes | iBMK parental and Akt cell lines | 3 | ^13^C and ^14^C isotope tracers | NMR; LC/MS; Isotope tracer | Sugar phosphate, amino acids, organic acids, redox factors, nucleotide related and coenzyme A and salts. | Glycolysis, PPP, TCA cycle | (26) |
| A valuable method for experimental elucidation of metabolic network topology is metabolic flux ratio analysis, which quantifies the relative contribution of two or more converging pathways to a given metabolite. It is based on ^13^C-labeling experiments, GC/MS, and probabilistic equations that relate mass distributions in proteinogenic amino acids to pathway activity. This describes the protocol for sample generation and illustrates the principles underlying the calculation of metabolic flux ratios with three examples. These principles are also implemented in the publicly available software FiatFlux, which directly calculates flux ratios from the mass spectra of amino acids | *Escherichia coli*  or  *Bacillus subtilis* | 3 | [1-^13^C] glucose | GC/MS; Analysis Software FiatFlux | Sugar phosphate, organic acids, redox factors, nucleotide related, coenzyme A and salts | Entner-Doudoroff (ED) pathway; PP pathway, pentose-phosphate (PPP) pathway and TCA cycle | (27) |
| Investigating control of metabolic flux in the model bacterium *Bacillus subtilis* by quantifying fluxes, transcripts, and metabolites in eight metabolic states enforced by different environmental conditions. This shows that most enzymes whose flux switches between on and off states, such as those involved in substrate uptake, exhibit large corresponding transcriptional changes. Also, substrate changes revealed by metabolomics are insufficient to explain observed fluxes, suggesting a large role for allosteric regulation and enzyme modification in the control of metabolic fluxes | *Bacillus subtilis* | 2 | ^13^C-labeling  Isotopes. | GC/MS | Sugar phosphate, amino acids, organic acids, redox factors, nucleotide related and coenzyme A and salts | Glycolysis, the pentose phosphate and TCA cycle | (28) |
| A combined approach (flux ratio and computational tools) to characterize the difference in metabolic flux of developing seeds of two *B. napus* genotypes contrasting in starch and oil content. | *Brassica napus L.; Brassicaceae* plants | 13 | ^13^C-labeling  Isotopes | GC/MS; ^13^CFLUX2 computational toolbox | Free soluble metabolites, amino acids, Sugar phosphate, organic acids, redox factors, nucleotide related, coenzyme A and salts | Glycolysis, pentosephosphate pathway (PPP), the TCA cycle and biosynthetic effluxes into protein, lipid and free soluble metabolites for accession 3231 | (29) |
| The fluxes of (1) exogenous nitrogen (N) assimilation and (2) remobilization of endogenous N from vegetative plant compartments were measured by ^15^N labeling during the seed-filling period in pea (*Pisum sativum)* | Pea plants | 17 plants, 70 seeds | ^15^N-labeled  Nutrients | ^15^N-labeled plant; CHN analyzer | Net N accumulation forms | Exogenous and endogenous N pathways | (30) |
| Techniques based on linear programming to demonstrate   potential metabolic modules and other dynamics at the cellular level | *Arabidopsis thaliana* | 3 | Untargeted labelling | HPLC; Reverse-phase C18 column | Sugar phosphate, amino acids, organic acids, redox factors, nucleotide related and coenzyme A and salts | Pentose phosphate pathway,  CO_2_ fixation, Calvin cycle reactions, glycolysis and mitochondrial reactions | (31) |
| Measurement of the metabolic flux of glycogen biosynthesis using a dynamic metabolomic approach. Conclusive evidence of temporal alterations in the metabolic profile in cyanobacterial cells | *Arthrospira platensis* is (halophilic cyanobacterium) | N/A | ^13^C-labelling | HPLC; Sigma QuantiPro BCA Assay Kit; Capillary electrophoresis/mass spectrometry (CE/MS); Liquid chromatography/ triple quadrupole mass spectrometry (LC/QqQ-MS) | Sugar phosphates | Nonspecific metabolic pathways | (32) |
| Demonstrating a model of pyruvate distribution in *Lactococcus lactis* based on enzyme kinetics in combination with metabolic control analysis clearly indicates the key control points in the flux to acetoin and diacetyl, important flavor compounds. The model presented here showed that the enzymes with the greatest effect on this flux resided outside the acetolactate synthase branch itself | *Lactococcus lactis* | N/A | Fluorescein-labelled universal M13 primers | HPLC; Kinetic models | Sugar phosphate, amino acids, organic acids, redox factors, nucleotide related and coenzyme A and salts | Pentose phosphate pathway (PPP) | (33) |
| Preparing kinetic models including the reactions ranging from xylose transport into the cell to the phosphorylation of xylulose | *Cachromyaccharomyces cerevi* | N/A | Untargeted metabolomics. | Kinetic models (R-XDH and the XI models); HPLC | Xylulose-5-P, Xylitol | Xylose [reductase](https://www.sciencedirect.com/topics/biochemistry-genetics-and-molecular-biology/reductase)  (XR), xylitol [dehydrogenase](https://www.sciencedirect.com/topics/biochemistry-genetics-and-molecular-biology/dehydrogenase) (XDH) pathways | (34) |
| Estimating fluxes in the framework of kinetic flux profiling. Plants photosynthesizing under limiting irradiance and ambient CO_2_ in a custom-built chamber were transferred into a ^13^CO_2_-enriched environment. The isotope labeling patterns of 40 metabolites were obtained using LC-MS or GC-MS | *Arabidopsis thaliana* | 30 | ^13^CO_2_ Labeling Kinetics | LC-MS/MS | Sugar phosphate, amino acids, organic acids, redox factors, nucleotide related and coenzyme A and salts | Calvin-Benson Cycle, Starch, Sucrose, and Trehalose, Biosynthesis pathways | (35) |
| A comprehensive account of metabolomics and related NMR developments using 1-D NMR for metabolite profiling where compositional changes in a suite of metabolites are determined. It is useful to concentrate the extracts to be studied by susceptibility-matched volume limiting NMR tubes such as Shigemi tubes or small-volume probes such as a microprobe when dealing with samples of limited quantities such as biopsies or cell cultures, provided that the salt content is compatible. Although much NMR-based metabolomics research has been carried out in 1-D and 2-D, there are cases where 3-D experiments are called for, such as identification of metabolites which gather in a narrow spectral region | Mammalian cells | N/A | Isotopic labels (e.g., ^13^C, ^15^N, or ^2^H) | NMR (1 and 2-D) | Sugar phosphate, amino acids, organic acids, redox factors, fatty acids, nucleotide related and coenzyme A and salts | Glycolysis, TCA, cycle, fatty acid biosynthesis, pentose phosphate pathways | (36) |
| Understanding of the regulation of plant cell wall precursor metabolism using metabolic flux analysis based on dynamic labeling experiments. Arabidopsis T87 cells were cultured heterotrophically with ^13^C labeled sucrose. A kinetic model based on mass action reaction mechanisms was developed to simulate the carbon flow in the cell wall synthesis network. Kinetic parameters of the model were determined by fitting the model to the labeling time course data, cell wall composition, and synthesis rates | *Arabidopsis thaliana* | N/A | ^13^C labeled sucrose | GC/MS | Sugar phosphate, organic acids, redox factors, nucleotide related and coenzyme A and salts | [Oxidative pentose phosphate pathway](https://www.sciencedirect.com/topics/biochemistry-genetics-and-molecular-biology/pentose-phosphate-pathway) (OPPP) and [glycolysis](https://www.sciencedirect.com/topics/biochemistry-genetics-and-molecular-biology/glycolysis) | (37) |
| Introducing a genome-scale model for a plant with direct applications to food and bioenergy production (i.e., maize). The developed model corresponds to the largest and most complete to-date effort at cataloguing metabolism for a plant species | *Zea maize* | N/A | Untargeted metabolomics. | Computational models; Isotope labelling | Sugar phosphate, amino acids, organic acids, redox factors, fatty acids, nucleotide related and coenzyme A and salts | Primary (i.e., glycolysis, TCA, fatty acid and amino acid biosynthesis, starch and sucrose metabolism) and secondary (i.e., biosynthesis of steroid, ubiquionone, streptomycin, thiamin, riboflavin, terpenoid, brassinosteroid, phenylpropanoid, etc.) metabolism | (38) |
| A web-based platform MFlux ([http://mflux.org](http://mflux.org/)) that predicts bacterial central metabolism via machine learning, leveraging data from approximately 100 ^13^C-MFA papers on heterotrophic bacterial metabolisms combined with three machine learning methods | *Bacillus* species | 600 | ^13^C metabolic flux analysis (^13^C-MFA),  ^13^C isotopic labeling | NMR; ^13^C metabolic flux analysis (^13^C-MFA); Support vector machine (SVM), k-Nearest neighbors (k-NN), and decision tree | Sugar phosphate, amino acids, organic acids, redox factors, fatty acids, nucleotide related and coenzyme A and salts | Glycolysis, the tricarboxylic acid (TCA) cycle, the pentose phosphate (PP) pathway, the Entner-Doudoroff (ED) pathway, the glyoxylate shunt and the anaplerotic pathway | (39) |
| Ultraperformance liquid chromatography–time-of-flight-mass spectrometry (UPLC–TOFMS) was used to investigate sex specific differences in the mussel metabolome in order to further investigate the reproductive physiology of this species | *Mytilus edulis* | 40 | Labelled glycerol | UPLC–TOFMS | Glycerophosphatidylcholine (PC) and lysophosphatidyl choline (LPC) metabolites | Metabolic pathways associated with reproductive development | (40) |
| Using transcript profiling, which is supported by with relevant mutants, revealed transient changes during the adaptation to aerobic conditions | *E. coli* | N/A | Nucleotide fragments of target genes | Transcript profile | Lactate, succinate, ethanol, pyruvate, coenzyme A and acetate | Glycolysis | (41) |
| Investigating transcriptome dynamics during the transition from aerobic to micro-aerobic using transcript profiling | *E. coli* | N/A | labeled using Cy3-dCTP or Cy5-dCTP | Transcript profile | Sugar phosphate, amino acids, organic acids, redox factors, fatty acids, nucleotide related and coenzyme A and salts | Glycolysis, TCA, cycle, fatty acid biosynthesis, pentose phosphate pathways | (42) |
| Using ^2^H-labeled tracers enabled dissection of NADPH production routes across cell types and environmental conditions | HEK293T cells | N/A | ^2^H-labeled tracers | Isotopic tracing; RP-IP-LC-MS/MS; MALDI-ToF | Fatty acids, sugar phosphate, amino acids, organic acids, redox factors, nucleotide related and coenzyme A and salts | Hypoxia, NADPH  pathways | (43) |
| Using isotope tracers, mass spectrometry, and quantitative flux modeling, to directly map the metabolic pathways of *Clostridium acetobutylicum*, bacterium whose major fermentation products include the biofuels butanol and hydrogen | *C. acetobutylicum* | N/A | [U-^13^C] glucose | Isotope tracers; kinetic flux profiling (KFP); LC-ESI; RP-IP-LC-MS/MS | Sugar phosphate, organic acids, redox factors, nucleotide related and coenzyme A and salts | TCA cycle | (44) |
| The deletion of nfnAB and characterizing its role in *T.* *saccharolyticum* metabolism | *T. saccharolyticum* | 16 | ^13^C labeling | TOC-V CPH Elemental analyzer; HPLC; Biochemical assays | Xylose, ethanol, NADPH. | NADH-linked ethanol production | (45) |
| A protocol for analyzing incorporation of the non-radioactive stable isotopes carbon-13 (13C) and nitrogen-15 (15N) into polar metabolites in central carbon metabolism and related pathways | Mammalian cells | N/A | ^13^C/^15^N labelling | HPLC; UFLC; QTRAP-MS | Nucleotide related and coenzyme A Sugar phosphate, amino acids, organic acids, redox factors, fatty acids, and salts | Glycolysis, TCA, cycle, fatty acid biosynthesis, pentose phosphate pathways | (46) |
| Measuring the steady-state ratio of [1-^13^C] to [5-^13^C] glutamate using isotope tracing compared with other previous methods | Mammalian cells | N/A | [1-^13^C] acetate | Isotope tracers; NMR | Sugar phosphate, organic acids, redox factors, nucleotide related and coenzyme A and salts | TCA cycle | (47) |
| Using uniform labeled proteins in eukaryotic expression systems for structural biology, namely S*podoptera frugiperda* insect cells by applying isotope labelling and HPLC | Insect cells | 16 | Isotope labeling with 2H, 13C and 15N | Isotope tracers; HPLC-UV215; HPLC | Asparagine synthetase, glutamine synthetase, aspartate transaminase, glutamate dehydrogenase,  alanine transaminase | Residual H–N–CA pathway | (48) |
| Detecting the change in labeled substrate levels in both mutant TC265 and wild type to reveal the role of the chloroplast glucose transporter | *Arabidopsis thaliana* | 30 | [U-^14^C] glucose and [U-^14^C] glycerol | Labelled substrate | Glucose, sucrose and starch | Nonspecific metabolic pathways | (49) |
| A proof of principle study on human cancer cells with deep labeling to identify hundreds of endogenous metabolites as well as active and inactive pathways | HCT116 cancer cells | N/A | Different labelling isotopes | LC-HRMS; HPLC; MS; Labelling isotopes | Nucleotide related and coenzyme A Sugar phosphate, amino acids, organic acids, redox factors, fatty acids, and salts | Glycolysis, the pentose phosphate pathway, the tricarboxylic acid (TCA) cycle, amino acid metabolism, as well as de novo purine and pyrimidine synthesis | (50) |
| Isotopic nonstationary ^13^C flux analysis to map photoautotrophic fluxes in a terrestrial plant system. Alterations in photosynthetic carbon flux in response to high light acclimation. We provide a quantitative description of metabolism that accommodates acclimation and estimates changes in important fluxes that are difficult to measure. A comprehensive approach to map the flow and fate of carbon within plant metabolic networks | *Arabidopsis thaliana* | 10 | ^13^C-labeling patterns | Gas Exchange; ^13^CO_2_ Labeling; LC-MS/MS; GC-MS; Isotopomer Network and Flux Determination | Sugar phosphate, amino acids, organic acids, redox factors, fatty acids. | CBB cycle, photorespiration, a bifurcated TCA pathway, and pathways for starch, sucrose, and amino acid biosynthesis | (51) |
| Development of a microscale method to quantitatively assess hepatic glucose and intermediary metabolism in conscious, unrestrained mice. Performing GC-MS and mass isotopomer distribution (MID) analysis. This approach can accommodate a broad range of modeling assumptions, isotope tracers, and measurement inputs without the need to introduce ad hoc mathematical approximations | Male C57BL/6J mice | 24 | [^13^C_3_] propionate [^2^H_2_] water, and [6,6-^2^H_2_] glucose isotopes | GC-MS Analysis  Metabolic Flux Analysis | Nucleotide related and coenzyme A Sugar phosphate, amino acids, organic acids, redox factors, fatty acids, and salts. | CAC and glucose-producing pathways | (52) |
| Development of experimental conditions which closely mimic the postprandial state, the insulin resistant mouse heart retains the ability to stimulate glucose metabolism | Mice | 29 | ^13^C labelling | ^13^C labelling patterns, termed mass isotopomer distributions (MIDs) | Coenzyme A Sugar phosphate, amino acids, organic acids, redox factors, fatty acids, and salts, Nucleotide related and coenzyme A Sugar phosphate | Glycolytic and TCA cycle | (53) |
| Estimating the intracellular carbon flux distribution in wild-type and pyruvate kinase-deficient *Escherichia coli,* using biosynthetically directed fractional ^13^C labeling experiments with [U-^13^C_6_] glucose in glucose- or ammonia-limited chemostats, two-dimensional nuclear magnetic resonance (NMR) spectroscopy of cellular amino acids, and a comprehensive isotopomer model. Reliability of the flux estimates thus obtained was verified by statistical error analysis and by comparison to intracellular carbon flux ratios that were independently calculated from the same NMR data by metabolic flux ratio analysis. | *E. coli* | N/A |  | NMR; HPLC; METAFoR analysis |  | Pentose phosphate pathway (PPP) and the tricarboxylic acid (TCA) cycle. | (54) |

1. Mairinger T, Sanderson J, Hann S. GC-QTOFMS with a low-energy electron ionization source for advancing isotopologue analysis in (13)C-based metabolic flux analysis. Anal Bioanal Chem. 2019;411(8):1495-502.

2. Bergès C, Cahoreau E, Millard P, Enjalbert B, Dinclaux M, Heuillet M, et al. Exploring the Glucose Fluxotype of the E. coli y-ome Using High-Resolution Fluxomics. Metabolites. 2021;11(5):271.

3. Xiong W, Jiang H, Maness P. Dynamic Flux Analysis: An Experimental Approach of Fluxomics. Methods Mol Biol. 2020;2096:179-96.

4. Ahn E, Kumar P, Mukha D, Tzur A, Shlomi T. Temporal fluxomics reveals oscillations in TCA cycle flux throughout the mammalian cell cycle. Mol Syst Biol. 2017;13(11):953.

5. Schätzlein MP, Becker J, Schulze-Sünninghausen D, Pineda-Lucena A, Herance JR, Luy B. Rapid two-dimensional ALSOFAST-HSQC experiment for metabolomics and fluxomics studies: application to a (13)C-enriched cancer cell model treated with gold nanoparticles. Anal Bioanal Chem. 2018;410(11):2793-804.

6. Sá JV, Kleiderman S, Brito C, Sonnewald U, Leist M, Teixeira AP, et al. Quantification of Metabolic Rearrangements During Neural Stem Cells Differentiation into Astrocytes by Metabolic Flux Analysis. Neurochem Res. 2017;42(1):244-53.

7. Nix C, Hemmati M, Cobraiville G, Servais A-C, Fillet M. Blood Microsampling to Monitor Metabolic Profiles During Physical Exercise. Frontiers in Molecular Biosciences. 2021;8(423).

8. Huang Z, Xu J, Yongky A, Morris CS, Polanco AL, Reily M, et al. CHO cell productivity improvement by genome-scale modeling and pathway analysis: Application to feed supplements. Biochemical Engineering Journal. 2020;160:107638.

9. Katzir R, Polat IH, Harel M, Katz S, Foguet C, Selivanov VA, et al. The landscape of tiered regulation of breast cancer cell metabolism. Sci Rep. 2019;9(1):17760.

10. Cocuron J-C, Koubaa M, Kimmelfield R, Ross Z, Alonso AP. A Combined Metabolomics and Fluxomics Analysis Identifies Steps Limiting Oil Synthesis in Maize Embryos. Plant Physiology. 2019;181(3):961.

11. Weiner HS, Crosier AE, Keefer CL. Analysis of metabolic flux in felid spermatozoa using metabolomics and 13C-based fluxomics†. Biology of Reproduction. 2019;100(5):1261-74.

12. Wu C, Herold RA, Knoshaug EP, Wang B, Xiong W, Laurens LML. Fluxomic Analysis Reveals Central Carbon Metabolism Adaptation for Diazotroph Azotobacter vinelandii Ammonium Excretion. Scientific Reports. 2019;9(1):13209.

13. Lee SC, Shestov AA, Guo L, Zhang Q, Roman JC, Liu X, et al. Metabolic Detection of Bruton's Tyrosine Kinase Inhibition in Mantle Cell Lymphoma Cells. Mol Cancer Res. 2019;17(6):1365-77.

14. Carinhas N, Duarte TM, Barreiro LC, Carrondo MJ, Alves PM, Teixeira AP. Metabolic signatures of GS-CHO cell clones associated with butyrate treatment and culture phase transition. Biotechnol Bioeng. 2013;110(12):3244-57.

15. Lee WD, Mukha D, Aizenshtein E, Shlomi T. Spatial-fluxomics provides a subcellular-compartmentalized view of reductive glutamine metabolism in cancer cells. Nature Communications. 2019;10(1):1351.

16. Kim IY, Park S, Kim Y, Chang Y, Choi CS, Suh SH, et al. In Vivo and In Vitro Quantification of Glucose Kinetics: From Bedside to Bench. Endocrinol Metab (Seoul). 2020;35(4):733-49.

17. Cortassa S, Caceres V, Tocchetti CG, Bernier M, de Cabo R, Paolocci N, et al. Metabolic remodelling of glucose, fatty acid and redox pathways in the heart of type 2 diabetic mice. J Physiol. 2020;598(7):1393-415.

18. Bachhar A, Jablonsky J. A new insight into role of phosphoketolase pathway in Synechocystis sp. PCC 6803. Scientific Reports. 2020;10(1):22018.

19. Currin-Ross D, Husdell L, Pierens GK, Mok NE, O'Neill SL, Schirra HJ, et al. The Metabolic Response to Infection With Wolbachia Implicates the Insulin/Insulin-Like-Growth Factor and Hypoxia Signaling Pathways in Drosophila melanogaster. Frontiers in Ecology and Evolution. 2021;9(158).

20. Niittylae T, Chaudhuri B, Sauer U, Frommer WB. Comparison of quantitative metabolite imaging tools and carbon-13 techniques for fluxomics. Methods in molecular biology (Clifton, NJ). 2009;553:355-72.

21. Massou S, Nicolas C, Letisse F, Portais JC. NMR-based fluxomics: quantitative 2D NMR methods for isotopomers analysis. Phytochemistry. 2007;68(16-18):2330-40.

22. Hui S, Cowan AJ, Zeng X, Yang L, TeSlaa T, Li X, et al. Quantitative Fluxomics of Circulating Metabolites. Cell Metabolism. 2020;32(4):676-88.e4.

23. Saborano R, Eraslan Z, Roberts J, Khanim FL, Lalor PF, Reed MAC, et al. A framework for tracer-based metabolism in mammalian cells by NMR. Scientific Reports. 2019;9(1):2520.

24. Ragavan M, Merritt ME. Nuclear Magnetic Resonance Measurement of Metabolic Flux Using 13C and 1H Signals. In: Bhattacharya SK, editor. Metabolomics: Methods and Protocols. New York, NY: Springer New York; 2019. p. 29-40.

25. Dinclaux M, Cahoreau E, Millard P, Létisse F, Lippens G. Increasing field strength versus advanced isotope labeling for NMR-based fluxomics. Magn Reson Chem. 2020;58(4):305-11.

26. Fan J, Ye J, Kamphorst JJ, Shlomi T, Thompson CB, Rabinowitz JD. Quantitative flux analysis reveals folate-dependent NADPH production. Nature. 2014;510(7504):298-302.

27. Nanchen A, Fuhrer T, Sauer U. Determination of metabolic flux ratios from 13C-experiments and gas chromatography-mass spectrometry data: protocol and principles. Methods Mol Biol. 2007;358:177-97.

28. Chubukov V, Uhr M, Le Chat L, Kleijn RJ, Jules M, Link H, et al. Transcriptional regulation is insufficient to explain substrate-induced flux changes in Bacillus subtilis. Mol Syst Biol. 2013;9:709.

29. Hay JO, Shi H, Heinzel N, Hebbelmann I, Rolletschek H, Schwender J. Integration of a constraint-based metabolic model of Brassica napus developing seeds with (13)C-metabolic flux analysis. Front Plant Sci. 2014;5:724.

30. Schiltz S, Munier-Jolain N, Jeudy C, Burstin J, Salon C. Dynamics of exogenous nitrogen partitioning and nitrogen remobilization from vegetative organs in pea revealed by 15N in vivo labeling throughout seed filling. Plant Physiol. 2005;137(4):1463-73.

31. Poolman MG, Miguet L, Sweetlove LJ, Fell DA. A Genome-Scale Metabolic Model of Arabidopsis and Some of Its Properties    Plant Physiology. 2009;151(3):1570-81.

32. Hasunuma T, Kikuyama F, Matsuda M, Aikawa S, Izumi Y, Kondo A. Dynamic metabolic profiling of cyanobacterial glycogen biosynthesis under conditions of nitrate depletion. Journal of Experimental Botany. 2013;64(10):2943-54.

33. Hoefnagel MHN, Starrenburg MJC, Martens DE, Hugenholtz J, Kleerebezem M, Van Swam II, et al. Metabolic engineering of lactic acid bacteria, the combined approach: kinetic modelling, metabolic control and experimental analysisThe GenBank accession number for the sequence reported in this paper is AY046926. Microbiology. 2002;148(4):1003-13.

34. Parachin NS, Bergdahl B, van Niel EW, Gorwa-Grauslund MF. Kinetic modelling reveals current limitations in the production of ethanol from xylose by recombinant Saccharomyces cerevisiae. Metab Eng. 2011;13(5):508-17.

35. Szecowka M, Heise R, Tohge T, Nunes-Nesi A, Vosloh D, Huege J, et al. Metabolic fluxes in an illuminated Arabidopsis rosette. Plant Cell. 2013;25(2):694-714.

36. Fan TWM, Lane AN. Structure-based profiling of metabolites and isotopomers by NMR. Progress in Nuclear Magnetic Resonance Spectroscopy. 2008;52(2):69-117.

37. Chen X, Alonso AP, Shachar-Hill Y. Dynamic metabolic flux analysis of plant cell wall synthesis. Metab Eng. 2013;18:78-85.

38. Saha R, Suthers PF, Maranas CD. Zea mays iRS1563: A Comprehensive Genome-Scale Metabolic Reconstruction of Maize Metabolism. PLOS ONE. 2011;6(7):e21784.

39. Wu SG, Wang Y, Jiang W, Oyetunde T, Yao R, Zhang X, et al. Rapid Prediction of Bacterial Heterotrophic Fluxomics Using Machine Learning and Constraint Programming. PLOS Computational Biology. 2016;12(4):e1004838.

40. Cubero-Leon E, Minier C, Rotchell JM, Hill EM. Metabolomic analysis of sex specific metabolites in gonads of the mussel, Mytilus edulis. Comparative Biochemistry and Physiology Part D: Genomics and Proteomics. 2012;7(2):212-9.

41. Partridge JD, Scott C, Tang Y, Poole RK, Green J. Escherichia coli Transcriptome Dynamics during the Transition from Anaerobic to Aerobic Conditions. Journal of Biological Chemistry. 2006;281(38):27806-15.

42. Partridge JD, Sanguinetti G, Dibden DP, Roberts RE, Poole RK, Green J. Transition of Escherichia coli from Aerobic to Micro-aerobic Conditions Involves Fast and Slow Reacting Regulatory Components. Journal of Biological Chemistry. 2007;282(15):11230-7.

43. Liu L, Shah S, Fan J, Park JO, Wellen KE, Rabinowitz JD. Malic enzyme tracers reveal hypoxia-induced switch in adipocyte NADPH pathway usage. Nature Chemical Biology. 2016;12(5):345-52.

44. Amador-Noguez D, Feng X-J, Fan J, Roquet N, Rabitz H, Rabinowitz Joshua D. Systems-Level Metabolic Flux Profiling Elucidates a Complete, Bifurcated Tricarboxylic Acid Cycle in Clostridium acetobutylicum. Journal of Bacteriology. 2010;192(17):4452-61.

45. Lo J, Zheng T, Olson Daniel G, Ruppertsberger N, Tripathi Shital A, Guss Adam M, et al. Deletion of nfnAB in Thermoanaerobacterium saccharolyticum and Its Effect on Metabolism. Journal of Bacteriology. 2015;197(18):2920-9.

46. Yuan M, Kremer DM, Huang H, Breitkopf SB, Ben-Sahra I, Manning BD, et al. Ex vivo and in vivo stable isotope labelling of central carbon metabolism and related pathways with analysis by LC–MS/MS. Nature Protocols. 2019;14(2):313-30.

47. Burgess SC, Merritt ME, Jones JG, Browning JD, Sherry AD, Malloy CR. Limitations of detection of anaplerosis and pyruvate cycling from metabolism of [1-13C] acetate. Nature Medicine. 2015;21(2):108-9.

48. Sitarska A, Skora L, Klopp J, Roest S, Fernández C, Shrestha B, et al. Affordable uniform isotope labeling with (2)H, (13)C and (15)N in insect cells. J Biomol NMR. 2015;62(2):191-7.

49. Trethewey RN, ap Rees T. The role of the hexose transporter in the chloroplasts of Arabidopsis thaliana L. Planta. 1994;195(2):168-74.

50. Grankvist N, Watrous JD, Lagerborg KA, Lyutvinskiy Y, Jain M, Nilsson R. Profiling the Metabolism of Human Cells by Deep 13C Labeling. Cell Chemical Biology. 2018;25(11):1419-27.e4.

51. Ma F, Jazmin LJ, Young JD, Allen DK. Isotopically nonstationary 13C flux analysis of changes in Arabidopsis thaliana leaf metabolism due to high light acclimation. Proceedings of the National Academy of Sciences. 2014;111(47):16967-72.

52. Hasenour CM, Wall ML, Ridley DE, Hughey CC, James FD, Wasserman DH, et al. Mass spectrometry-based microassay of 2H and 13C plasma glucose labeling to quantify liver metabolic fluxes in vivo. American Journal of Physiology-Endocrinology and Metabolism. 2015;309(2):E191-E203.

53. Kowalski GM, De Souza DP, Risis S, Burch ML, Hamley S, Kloehn J, et al. In vivo cardiac glucose metabolism in the high-fat fed mouse: Comparison of euglycemic–hyperinsulinemic clamp derived measures of glucose uptake with a dynamic metabolomic flux profiling approach. Biochemical and Biophysical Research Communications. 2015;463(4):818-24.

54. Emmerling M, Dauner M, Ponti A, Fiaux J, Hochuli M, Szyperski T, et al. Metabolic Flux Responses to Pyruvate Kinase Knockout in Escherichia coli. Journal of Bacteriology. 2002;184(1):152-64.
